# Plasma calprotectin as a biomarker of mortality at antiretroviral treatment initiation in advanced HIV – pilot study

**DOI:** 10.12688/wellcomeopenres.15563.2

**Published:** 2020-11-27

**Authors:** Faith W. Kamau, Agnes Gwela, Andrew K. Nyerere, Victor Riitho, James M. Njunge, Moses M. Ngari, Andrew J. Prendergast, James A. Berkley

**Affiliations:** 1 Clinical Research, KEMRI/Wellcome Trust Research Programme, Kilifi, Kilifi County, 320-80108, Kenya; 2 Department of Molecular Biology and Biotechnology, Pan African University Institute for Basic Sciences, Technology and Innovation, Juja, Nairobi, 62000-00200, Kenya; 3 Childhood Acute Illness & Nutrition (CHAIN) Network, Nairobi, Nairobi, 43640-00100, Kenya; 4 Department of Medical Microbiology, Jomo Kenyatta University of Agriculture and Technology, Juja, Nairobi, 62000–00200, Kenya; 5 Blizard Institute, Queen Mary University of London, London, London, E1 2AT, UK; 6 Centre for Tropical Medicine & Global Health, University of Oxford, Oxford, Oxfordshire, OX3 7FZ, UK

**Keywords:** HIV, CD4, Mortality; Adult, Neutrophil, Antiretroviral, Biomarker, Prognostic

## Abstract

**Background:** In advanced HIV, significant mortality occurs soon after starting antiretroviral treatment (ART) in low- and middle-incomes countries. Calprotectin is a biomarker of innate response to infection and inflammatory conditions. We examined the association between plasma calprotectin collected before ART treatment and mortality among individuals with advanced HIV. **Methods: **We conducted a pilot case-cohort study among HIV infected adults and adolescents over 13 years old with CD4+ <100/mm3 at ART initiation at two Kenyan sites. Participants received three factorial randomised interventions in addition to ART within the REALITY trial (ISRCTN43622374). Calprotectin collected at baseline (before ART) and after 4 weeks of treatment was measured in archived plasma of those who died within 24 weeks (cases) and randomly selected participants who survived (non-cases). Association with mortality was assessed using Cox proportional hazards models with inverse sampling probability weights and adjusted for age, sex, site, BMI, viral load, randomised treatments, and clustered by CD4+ count (0-24, 25-49, and 50-99 cells/mm3).**** **Results: **Baseline median (IQR) plasma calprotectin was 6.82 (2.65–12.5) µg/ml in cases (n=39) and 5.01 (1.92–11.5) µg/ml in non-cases (n=58). Baseline calprotectin was associated with age, neutrophil count and the presence of cough, but not other measured indicators of infection. In adjusted multivariable models, baseline calprotectin was associated with subsequent mortality: HR 1.64 (95% CI 1.11 - 2.42) and HR 2.77 (95% CI 1.58 - 4.88) for deaths during the first twenty-four and four weeks respectively. Calprotectin levels fell between baseline and 4 weeks among both cases and non-cases irrespective of randomised interventions. **Conclusions:** Among individuals with advanced HIV starting ART in Kenya, plasma calprotectin may have potential as a biomarker of early mortality. Validation in larger studies, comparison with other biomarkers and investigation of the sources of infection and inflammation are warranted.

## Introduction

Approximately one-quarter of individuals newly diagnosed with HIV in sub-Saharan Africa have advanced disease at presentation ( IeDEA and ART Cohort Collaborations *et al*., 2014). Advanced HIV is characterized by immunosuppression, infection and immune activation which may independently drive mortality despite antiretroviral therapy (ART). Measurements of soluble biomarkers such as soluble CD14, C-reactive protein and IL-18 have highlighted that inflammation and innate immune responses predict all-cause mortality, cardiovascular events, and other morbidities in HIV infected individuals, even after starting ART treatment ( Duprez *et al*., 2012; Kuller *et al*., 2008; Sandler & Douek, 2012). Overall, innate immune activation seems more important than T-cell activation for disease progression in sub-Saharan Africa ( Hunt *et al.*, 2016; Serrano-Villar *et al*., 2014). Inflammation and co-infection can also arise from disruption of intestinal tight junctions leading to increased mucosal permeability. Translocation from the intestine of bacteria and their products including lipopolysaccharides has been demonstrated in some ( Brenchley *et al*., 2006; Marchetti *et al.*, 2013; Nazli *et al*., 2010; Sandler & Douek, 2012), but not all studies ( Fitzgerald *et al*., 2019).

Calprotectin is a soluble 24 kDa dimer of calcium-binding proteins S100A8 and S100A9 ( Brophy & Nolan, 2015) produced by neutrophils and other cells following activation in response to infection and inflammation. Calprotectin, measured in either stool or plasma, is a recognised biomarker of inflammation and bacterial infections including sepsis ( Banerjee *et al*., 2015; Bjarnason, 2017; Huang *et al*., 2016; Jonsson *et al*., 2017; Simm *et al*., 2016; Walsham & Sherwood, 2016). The S100A9 sub-unit of plasma calprotectin has been associated with reduced immune reconstitution after ART ( Drozd *et al*., 2016), enhanced antimicrobial defence transiently induced by antiviral treatment ( Muller *et al*., 1994) and HIV-associated neurocognitive disorders ( Colon *et al*., 2016). Njunge *et al.* recently demonstrated an association between increased plasma calprotectin and early post-discharge mortality among HIV-uninfected children hospitalized for severe acute malnutrition ( Njunge *et al*., 2019).

We considered that calprotectin may be of value as a prognostic biomarker in advanced HIV and conducted a pilot study to evaluate associations between plasma calprotectin and mortality in individuals with advanced HIV infection prior to ART initiation who participated in the Reduction of Early Mortality in HIV Infected Adults and Children Starting Antiretroviral Therapy (REALITY) trial ( Hakim *et al*., 2017) ( Kityo *et al*., 2018) ( Mallewa *et al*., 2018).

## Methods

### Study population

The REALITY trial ( ISRCTN43622374) enrolled HIV-infected adults and children aged five years or more with a CD4 ^+^ T cell count <100 cells/mm ^3 ^and without previous ART treatment at 8 sites in Kenya, Uganda, Malawi, and Zimbabwe. Participants in the REALITY trial were enrolled between August, 2013 and April, 2015 and randomised to three factorial treatments compared to standard of care: enhanced antimicrobial prophylaxis (single-dose albendazole, 5 days of azithromycin, 12 weeks of fluconazole (100 mg), and 12 weeks of fixed-dose combination of cotrimoxazole (800/160 mg)/isoniazid (300 mg)/pyridoxine (25 mg) once daily) ( Hakim *et al*., 2017); additional raltegravir ( Kityo *et al*., 2018); and ready to use supplementary food (RUSF) ( Mallewa *et al*., 2018).

This pilot study capitalised on a broader ongoing immunology case-cohort sub-study that included study participants aged 13 years or more with a sample set of plasma, baseline stool, PBMCs and data at two Kenyan sites: Kilifi County Hospital and the Academic Model for the Prevention and Treatment of HIV/AIDS Centre at Moi Teaching Referral Hospital, Eldoret.

### Study design

**Figure 1.**
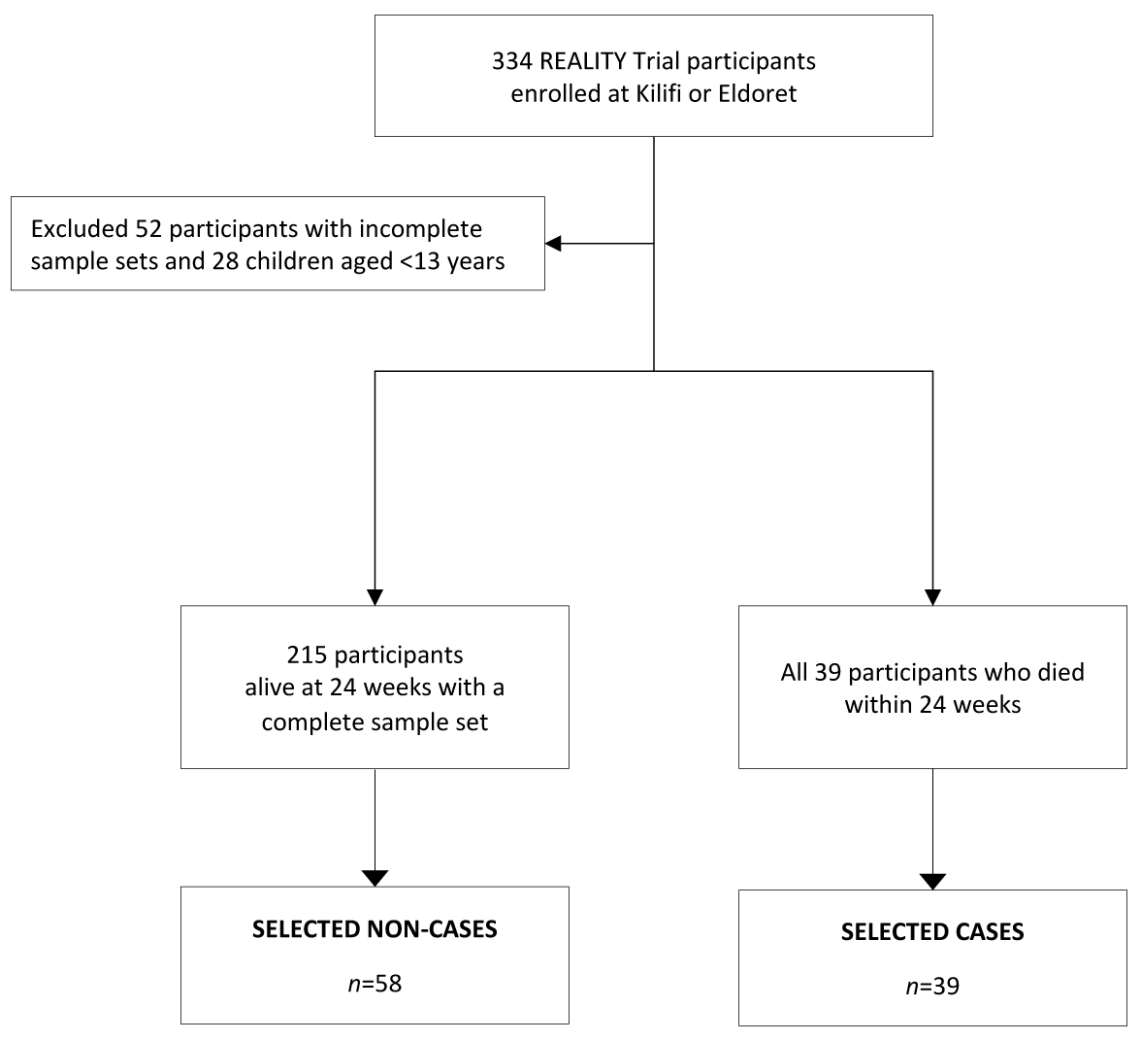
Participant selection.

This pilot was a case-cohort study. The REALITY trial had enrolled a total of 139 participants in Kilifi, of whom 29 (20%) died, and 195 in Eldoret, of whom 14 (7.2%) died ( Figure 1). However, in Kilifi baseline CD8 measurements were missing on approximately one-third of participants due to reagent unavailability at specific time periods (i.e., missing at random) and one-quarter were also missing stored specimens for similar reasons. Therefore, the required number of participants in Kilifi were selected from those with complete samples and baseline CD8 available to ensure that data could be obtained, and weighted (see below) to reflect the original trial population. The immunology case-cohort sub-study randomly selected 45% of all participants at Kilifi and 10% of study participants at Eldoret as a sub-cohort in order to reflect enrolment and mortality in the full REALITY trial, stratified by CD4 ^+^ count (0–24, 25–49, and 50–99 cells/mm ^3^) to avoid imbalance in this exposure, plus any remaining unselected deaths by week 24 (the trial primary endpoint). Non-cases were randomly selected from those who survived through 24 weeks from the trial database using the uniform random number function in STATA.

Deaths were weighted as 1 and non-deaths were weighted as 106/42 in Kilifi and 174/16 in Eldoret. These values were chosen in order that the sample represented the full trial population in terms of deaths and survivors at these sites using the inverse probability of selection from all REALITY patients ≥13 years of age alive at week 24 (regardless of immunology sub-study membership and available samples). Demographic, clinical and laboratory data were collected during the REALITY trial using standardised case report forms ( Hakim *et al*., 2017). Complete blood counts, including neutrophils and CD4 ^+^ counts were done at local laboratories. Samples analysed were those prior to receiving ART at baseline, then 4 weeks after starting ART.

### Enzyme-linked immunosorbent assay (ELISA)

Plasma calprotectin was measured in duplicate at recommended dilutions using a solid-phase enzyme-linked immunosorbent assay (ELISA) human calprotectin kit (Hycult Biotech HK379–02) according to the manufacturer’s instructions and the absorbance read at 450 nm using a Synergy 4 (BioTek) plate reader. Calprotectin was measured using samples collected at baseline, before ART treatment was initiated, and at four weeks after ART initiation. The four-week time period allowed an appropriate time frame for the detection of immunological changes following treatment initiation.

### Statistical analysis

Data were analysed using STATA version 13.1 (STATA corp. TX, USA). The baseline characteristics of the selected study participants were presented as mean (SD) for normally distributed variables, as median with interquartile range (IQR) for non-normally distributed variables, and as numbers (percentage) for categorical data. To compare differences in characteristics between cases and non-cases, Student’s *t*-test and Mann-Whitney rank-sum test (of non-normally distributed variables) were used. Chi-square or Fisher’s exact tests were used to compare proportions and Spearman's correlation to assess correlation.

Factors associated with plasma calprotectin levels at baseline were assessed using a stepwise linear regression model including age, sex, site, CD4 ^+^, viral load and neutrophil count, but excluding the randomised interventions. Individual clinical and laboratory features of infection with biological plausibility: the presence of fever, cough, diarrhoea, known tuberculosis, measured body temperature, CD8 ^+^ T cell count, and cryptococcal antigen test were then added one by one, retaining only those which were statistically significant at P<0.1. The beta coefficient indicated the strength of the effect on plasma calprotectin.

The association between baseline plasma calprotectin and mortality was assessed using a stratified Cox proportional hazards regression model with inverse probability weights ( Buchanan *et al*., 2014) with three strata by CD4 count (0–24, 25–49, and 50–99 cells/mm ^3^) to reflect the proportions selected in the case-cohort design to help address bias, as described above. Time at risk was defined from enrolment to 24 weeks which was the primary endpoint of the parent trial. Mortality within the first four weeks, when most deaths occurred, was also assessed. The hazard ratio per unit increase in calprotectin was estimated, after adjustment for age, sex, BMI, log viral load, site and each of the randomised treatment arms. Akaike (AIC) and Bayesian (BIC) information criteria were calculated to assess model performance.

The Wilcoxon paired sign-rank test was used to assess the change in plasma calprotectin between baseline and after four weeks following treatment. Generalised linear modelling, adjusted for age, sex and site and the three randomised interventions and stratified by CD4 ^+^ group as above was used to examine relative risk for subsequent mortality.

### Ethical considerations

The study protocol was approved by the Kenya Medical Research Institute (KEMRI) Scientific and Ethics Review Committee approval number SSC 2231. Written informed consent was obtained from all participants using local languages, which included permission for storage and testing for this work.

## Results

### Characteristics of study participants

Of the selected 97 participants, 39 were cases and were 58 non-cases ( Figure 1). The baseline characteristics of the participants are shown in Table 1. A total of 16/39 (41%) cases died between enrolment and four weeks, and a further 23/39 (59%) cases died between four and 24 weeks. Death occurred at a median of 31 (Interquartile range, IQR 18–72) days after enrolment. Cases had a lower BMI, haemoglobin and CD8 ^+^ T cell counts than non-cases, and fewer cases were randomized to receive RUSF in univariate analyses.

**Table 1.** Participant characteristics.

Characteristic	Cases (n=39)	Non-Cases (n=58)	P Value
**Site**			0.55
Kilifi (%)	26 (67)	42 (72)	
Eldoret (%)	13 (33)	16 (28)	
**Sex**			0.84
Male (%)	18 (46)	28 (48)	
Female (%)	21 (54)	30 (52)	
**Age (**years)	41 (7.5)	39 (10.5)	0.35
**BMI** (kg/m ^2^)	16.9 (3.5)	18.3 (2.7)	0.04
**Full blood count**			
Haemoglobin (g/dL)	9.3 (7.3 - 10.5)	9.9 (9.0 - 11.8)	0.03
CD4 ^+^ count (cells/mm ^3^)	22 (8 - 44)	21 (11 - 64)	0.33
CD8 ^+^ count (cells/mm ^3^)	377 (242 - 707)	646 (410 - 931)	0.02
Viral load (×10 ^3^/mL) (copies/mL)	255 (132 - 760)	254 (115 - 521)	0.80
Plasma Calprotectin (µg/mL)	6.82 (2.65 – 12.5)	5.01 (1.92 – 11.5)	0.23
Neutrophil count (×10 ^9^/L)	2.26 (1.29 - 3.66)	1.77 (1.26 - 2.71)	
**Antimicrobial prophylaxis**			0.43
Standard (%)	24 (62)	31 (53)	
Enhanced (%)	15 (38)	27 (47)	
**Additional raltegravir**			0.30
No Raltegravir (%)	18 (46)	33 (57)	
Raltegravir (%)	21 (54)	25 (43)	
**Nutritional supplement**			0.02
No RUSF (%)	12 (31)	32 (55)	
RUSF (%)	27 (69)	26 (45)	

Categorical data represented as number (percentage) and continuous data as mean (SD) for the normally distributed and as median (IQR) for the non-normally distributed. Abbreviations: BMI, body mass index; RUSF, ready to use supplementary food; CD, cluster of differentiation; HIV, human immunodeficiency virus.

### Baseline plasma calprotectin

At enrolment, unadjusted calprotectin levels were median (IQR) 6.82 (2.65 to 12.5) µg/mL in cases (n=39) and 5.01 (1.92 to 11.5) µg/ml in non-cases (n=58). Higher age was associated with lower plasma calprotectin at baseline (β=-0.02; 95% CI -0.40 to -0.01; P=<0.01) and there was positive association with female sex (β=-0.35; 95% CI 0.04 to 0.66; P=0.03), neutrophil count (β=-0.19; 95% CI 0.12 to 0.27; P=<0.01) and reporting a cough (β=-0.51; 95% CI -0.13 to 0.88; P=0.01) ( Table 2). Other putative markers of infection (CD8 ^+^ T cells, fever, tuberculosis, body temperature, diarrhoea, and cryptococcal antigen test) were not statistically significant when their effect on plasma calprotectin levels was tested.

**Table 2.** Multivariable stepwise linear regression analysis of non-randomised variables with log plasma calprotectin at baseline.

Variable	Beta with interpretation	95% CI	P value
Age per year	-0.02 (calprotectin is lower in older participants)	-0.40 to -0.01	<0.01
Sex (female)	0.35 (females had higher calprotectin than males)	0.04 to 0.66	0.03
Site (Eldoret)	-0.22 (calprotectin was lower in Eldoret than Kilifi)	-0.57 to 0.14	0.23
Log viral load	0.01 (viral load was not associated with calprotectin)	-0.13 to 0.10	0.86
CD4+ 50-99/mm ^3^	Reference		
CD4+ 25-49/mm ^3^	-0.76 (calprotectin did not vary by CD4 ^+^ strata)	-0.54 to 0.39	0.75
CD4+ 0-24/mm ^3^	0.06 (calprotectin did not vary by CD4 ^+^ strata)	-0.29 to 0.41	0.75
Neutrophils ×109/L	0.19 (calprotectin is positively associated with neutrophil count)	0.12 to 0.27	<0.01
Cough	0.51 (calprotectin was higher in patients with cough)	0.13 to 0.88	0.01

N=95. CD4 ^+^ T cell count was stratified into 0-24, 25-49, and 50-99 cells/mm ^3^. The following variables were tested but excluded: CD8+ T cells, fever, tuberculosis, body temperature, diarrhoea, and cryptococcal antigen test

### Association with mortality

Baseline calprotectin was significantly associated with subsequent mortality to 24 weeks (HR 1.82 (95% CI 1.08 to 3.08), *P*=0.03) and mortality within the first 4 weeks (HR 2.77 (95% CI 1.58 to 4.88), *P*<0.001) in multivariable models adjusted for potential confounders ( Table 3).

**Table 3.** Hazards ratios for mortality in the first 4 and 24 weeks.

	Deaths within 24 weeks		Deaths within 4 weeks	
Variable	HR [95% CI]	P	HR (95% CI)	P
Age per year	1.04 (0.99 - 1.09)	0.06	1.04 (0.98 – 1.11)	0.04
Sex (female)	0.84 (0.33 – 2.18)	0.73	0.29 (0.09 – 0.97)	0.19
BMI per kg/m ^2^	0.83 (0.67 - 1.01)	0.07	0.94 (0.76 - 1.18)	0.60
Log viral load	0.97 (0.57 - 1.63)	0.91	0.91(0.48 - 1.73)	0.77
Site (Eldoret)	0.70 (0.23 – 2.17)	0.29	0.35 (0.06 – 2.14)	0.25
Calprotectin per µg/ml	1.82 (1.08 – 3.08)	0.03	2.77 (1.58 - 4.88)	<0.001
Information criteria	AIC 87.9; BIC 111		AIC 41.9; BIC 65.0	

Cox proportional hazards model stratified by three CD4 count groups (0-24, 25-49, and 50-99 cells/mm ^3^) and adjusted for the three randomised interventions. BMI: Body Mass Index.

### Plasma calprotectin after 4 weeks

**Figure 2.**
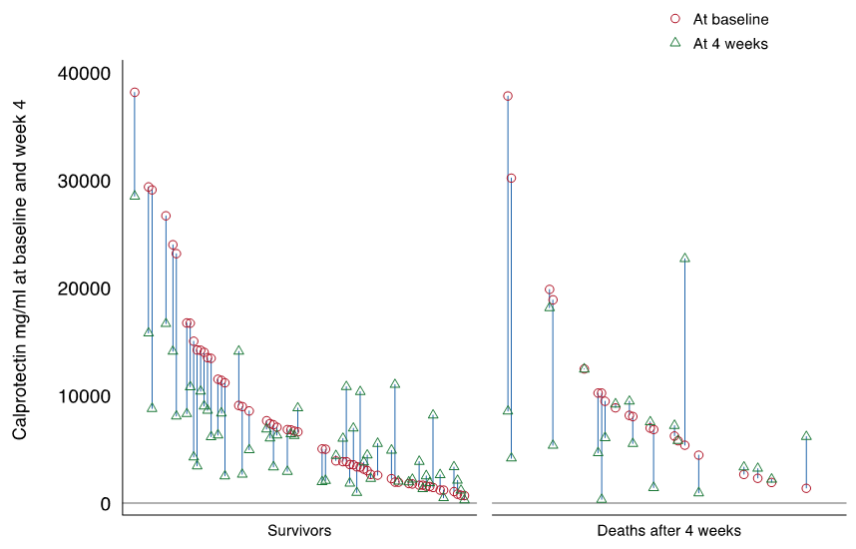
Plasma calprotectin at baseline and at four weeks. Participants sorted by baseline calprotectin values (high to low), N=78.

Between baseline and four weeks, calprotectin declined both in cases who were still alive at 4 weeks (n=21) and in non-cases (n=57), without evidence of difference between cases and non-cases ( Table 4). The three randomised interventions did not have statistically significant effects on the change in calprotectin between baseline and 4 weeks: additional raltegravir ß1.02 (95% CI -2.26 to 4.29); enhanced antimicrobial prophylaxis ß -0.55 (95% CI -3.86 to 2.77); ready-to-use supplementary food ß 2.18 (95% CI -1.153 to 5.52). Two cases who died after 4 weeks (10%) and four non-cases (7%) had a >2-fold rise in plasma calprotectin between baseline and 4 weeks ( Figure 2). Overall, change in plasma calprotectin between baseline and 4 weeks was not associated with subsequent mortality: RR 1.02 (95% CI 0.93 - 0.11) per µg/ml (P=0.664).

**Table 4.** Change in plasma calprotectin between baseline and four weeks.

	Median Calprotectin µg/ml at baseline	Median Calprotectin µg/ml at 4 weeks	Median * change from baseline to 4 weeks	IQR
**All participants alive at 4 weeks**	6.70	5.52	-0.58 *	-5.20 to +0.96
**Cases that died after 4 weeks**	8.03	5.78	-0.02	-5.40 to +0.68
**Non cases**	5.03	4.94	-0.72	-5.00 to +1.27

Only participants alive at 4 weeks are shown (N=78).

* For the change between baseline and 4 weeks in all participants P=0.005; change in cases vs. non-cases P=0.38 adjusted for age, sex, site and randomised interventions.

## Discussion

This pilot study focused on assessing the association with pre-ART plasma calprotectin and mortality within 24 weeks among patients with advanced HIV disease defined by a very low CD4 ^+^ cell count (<100cells/mm ^3^). Plasma calprotectin at the time of ART initiation was associated with subsequent mortality within 24 weeks as well as in the first 4 weeks. There was no evidence that the enhanced opportunistic infection intervention in the REALITY trial, which was associated with a 27% reduction in mortality to 24 weeks, ( Hakim *et al*., 2017) affected changes in calprotectin during the first 4 weeks. Interestingly, although none of the three interventions significantly affected the change in plasma calprotectin in this pilot study, the point estimate for enhanced antimicrobial prophylaxis, which was associated with reduced mortality in the parent trial ( Hakim *et al*., 2017) was in a negative direction. However, the sample size for this exploratory analysis was limited. This pilot study requires validation in a larger sample and in other clinical settings before being used to guide further investigations or specific interventions.

To the best of our knowledge, this is the first study looking at plasma calprotectin in the context of mortality in advanced HIV. Previous studies have focused on faecal calprotectin as a biomarker of enteropathy, which is typically elevated in HIV-positive compared to HIV-negative individuals and progressively increases with a reduction in CD4 ^+^ T cell count ( Hestvik *et al*., 2012). Faecal calprotectin is elevated in both early and chronic HIV infection and the elevated levels of faecal calprotectin have been associated with microbial translocation and enteropathy ( Pastor *et al*., 2019).

Our results indicated a significant positive correlation between plasma calprotectin and neutrophil counts at baseline which has been observed previously ( Cotoi *et al*., 2014; Sorensen *et al*., 2015; Sun *et al*., 2014) and likely reflects neutrophil expansion and activation as a main source of calprotectin ( Chatzikonstantinou *et al*., 2016). Plasma calprotectin may be a marker of systemic inflammation as a result of microbial translocation ( Deeks *et al*., 2013; Jonsson *et al*., 2017) or could indicate the presence of opportunistic infections among advanced HIV-positive patients, rather than only inflammation due to infection with HIV. However, markers of infection markers apart from cough were not statistically significantly associated with baseline plasma calprotectin. This could result due to the small number of samples tested, hence insufficient power.

The main strength of our study is that it was carried out in typical African hospital-based HIV clinics in which all eligible patients from both urban and peri-urban areas were recruited and is thus a reasonable reflection of advanced HIV patients in sub-Saharan Africa. Moreover, the use of ELISA is feasible in these setups, and already in application as a confirmatory test for HIV positive diagnosis. Besides the sample size of this pilot study, limitations included that despite weighting there was potential for bias during sample selection as included participants were required to have a full set of samples, which varied by site, and children under thirteen years old were excluded. The parent trial only enrolled patients with advanced disease, therefore we recommend that future studies be carried on patients with less advanced HIV to elucidate whether plasma calprotectin has similar predictive value, along with other proteins, metabolites and cytokines.

## Conclusions

Findings from this pilot study suggest that plasma calprotectin at the time of ART initiation has value in predicting early mortality among HIV patients with advanced disease. There is at least one quantitative serum calprotectin lateral-flow test available, and ELISA- or lateral flow-based tests may be useful in patient care. However, further validation of plasma calprotectin as a clinical tool is needed before incorporating the biomarker to guide enhanced investigation for infections, more frequent follow up or specific interventions.

## Data availability

### Underlying data

De-identified REALITY trial data are available from MRC CTU at UCL, which encourages optimal use of data by employing a controlled access approach to data sharing, incorporating a transparent and robust system to review requests and provide secure data access consistent with the relevant ethics committee approvals. All requests for data are considered and can be initiated by contacting mrcctu.ctuenquiries@ucl.ac.uk Quoting “REALITY Trial Immunology”.
