# Supplementary material for: Plasma calprotectin as a biomarker of mortality at antiretroviral treatment initiation in advanced HIV – pilot study
Source: Wellcome Open Res. 2020 Nov 27;5:46. Originally published 2020 Mar 13. [Version 2] doi: 10.12688/wellcomeopenres.15563.2 (PMC7722532; doi:10.12688/wellcomeopenres.15563.2)
Supplement: Peer review history

# Peer review: Reviewer response for version 2

**DOI:** 10.21956/wellcomeopenres.17976.r41660

**Published:** 2020-12-07

**Authors:** Rachel A. Silverman

**Affiliations:** 1 Center for Public Health Practice and Research, Department of Population Health Sciences, Virginia Polytechnic Institute and State University, Blacksburg, VA, USA

The authors have sufficiently addressed my initial comments. I have no further comments to make.

Is the work clearly and accurately presented and does it cite the current literature?

Yes

If applicable, is the statistical analysis and its interpretation appropriate?

Partly

Are all the source data underlying the results available to ensure full reproducibility?

Partly

Is the study design appropriate and is the work technically sound?

Partly

Are the conclusions drawn adequately supported by the results?

Partly

Are sufficient details of methods and analysis provided to allow replication by others?

Partly

Reviewer Expertise:

Epidemiology, HIV treatment and prevention, mortality following ART initiation. This topic was a focus of my recent dissertation work, though I have since switched to other topics related to other STDs and specific issues related to health in Southwest Virginia in the United States, so my knowledge on this specific topic may be a couple of years out of date.

I confirm that I have read this submission and believe that I have an appropriate level of expertise to confirm that it is of an acceptable scientific standard.

# Peer review: Reviewer response for version 2

**DOI:** 10.21956/wellcomeopenres.17976.r41659

**Published:** 2020-12-03

**Authors:** Rupak Shivakoti

**Affiliations:** 1 Columbia University Mailman School of Public Health, New York, NY, USA

The only optional comment for authors to consider is that Table 2, if there is sufficient power, could be analyzed using only the subcohort data. It would be taking advantage of the randomly selected subcohort vs. using the full case-cohort which was selected based on mortality (not the relevant outcome for Table 2). Overall, the authors have addressed earlier concerns and the new version of the manuscript is stronger and acceptable for indexing.

Is the work clearly and accurately presented and does it cite the current literature?

Yes

If applicable, is the statistical analysis and its interpretation appropriate?

Partly

Are all the source data underlying the results available to ensure full reproducibility?

Yes

Is the study design appropriate and is the work technically sound?

Partly

Are the conclusions drawn adequately supported by the results?

Yes

Are sufficient details of methods and analysis provided to allow replication by others?

Partly

Reviewer Expertise:

HIV; Inflammation; Epidemiology; HIV treatment outcomes.

I confirm that I have read this submission and believe that I have an appropriate level of expertise to confirm that it is of an acceptable scientific standard.

# Peer review: Reviewer response for version 1

**DOI:** 10.21956/wellcomeopenres.17044.r39054

**Published:** 2020-06-26

**Authors:** Rupak Shivakoti

**Affiliations:** 1 Columbia University Mailman School of Public Health, New York, NY, USA

This is a pilot study assessing the relationship of plasma calprotectin, a marker of inflammation, with mortality among individuals with advanced HIV initiating treatment. The authors use a nested case-cohort design to measure inflammation at time of ART initiation and use data from the parent study to assess mortality during treatment. Their results show that plasma calprotectin could be useful marker for subsequent mortality, especially within the next 4 weeks, in individuals with advanced HIV. This is a clearly written manuscript and the design seems appropriate. There are, however, a few major questions that should be addressed. 

 Comments:

- Given differences in immunity for children compared to adults, the percentage of children in this study should be specified and it would be helpful to know whether the baseline levels of calprotectin was different between children and adults. It is likely that the study is underpowered to look at association with mortality separately for children and adults but the authors should discuss whether combining them (including under 5 as suggested in discussion) in one analysis might or might not make sense for this specific marker.
- It is not clear why 45% of Kilifi participants vs. only 10% of Eldoret were selected for the random subcohort. It is also not clear how this case-cohort was selected from the larger immunology case-cohort.
- The case-cohort design has a random subcohort. How many cases out of 39 were part of the random subcohort? It is not clear from reading this what the sample size of the random subcohort is.
- In the methods, the authors say: “The linear regression to examine whether subsequent death and the 3 randomized interventions were associated with the change in plasma calprotectin between baseline and 4 weeks”
  - The mortality analysis is confusing as written. I don’t think the results were presented in the manuscript? For example, it is not clear what the exposure and outcome variable for this analysis is. If the exposure is change in plasma calprotectin, then the outcome is presumably death after 4 weeks (which would exclude many deaths) and a linear regression does not seem appropriate. But if the exposure is death (i.e. before 4 weeks), then a 4 week sample would not be available.
- Sample size should be stated for the tables and figures. Was the random subcohort used in any of the presented analysis? E.g. due to random selection, it may be most useful for Table 2 analysis if there are enough cases in the subcohort.
- Given the small sample size, selecting covariates for multivariable model based on significance on univariate models can present some issues. Lack of significance could be due to the limited power for this specific study although a real association may exist.
- Minor: It is useful to also see the hazards and 95% CI (not just p-values) in text of results instead of just the table.
- Discussion should include whether this marker could have utility for individuals with less advanced HIV.

Is the work clearly and accurately presented and does it cite the current literature?

Yes

If applicable, is the statistical analysis and its interpretation appropriate?

Partly

Are all the source data underlying the results available to ensure full reproducibility?

Yes

Is the study design appropriate and is the work technically sound?

Partly

Are the conclusions drawn adequately supported by the results?

Yes

Are sufficient details of methods and analysis provided to allow replication by others?

Partly

Reviewer Expertise:

HIV; Inflammation; Epidemiology; HIV treatment outcomes.

I confirm that I have read this submission and believe that I have an appropriate level of expertise to confirm that it is of an acceptable scientific standard, however I have significant reservations, as outlined above.

# Response

**Published:** 2020-09-29

**Authors:** James Berkley

We thank the reviewer for their comments providing an opportunity to improve our manuscript. Please find out point-by-point responses below.  

**Reviewer 2** Given differences in immunity for children compared to adults, the percentage of children in this study should be specified and it would be helpful to know whether the baseline levels of calprotectin was different between children and adults. It is likely that the study is underpowered to look at association with mortality separately for children and adults but the authors should discuss whether combining them (including under 5 as suggested in discussion) in one analysis might or might not make sense for this specific marker.  

*In the parent study, children recruited were 5-12 years, adolescents were 13-17 years and adults were 18 years and above. In this pilot sub-study we only included participants ≥ 13 years and above. As such, data from children is out of scope and not available for this plot study.*  

It is not clear why 45% of Kilifi participants vs. only 10% of Eldoret were selected for the random subcohort. It is also not clear how this case-cohort was selected from the larger immunology case-cohort.  

*The broader case cohort study involved two of the sites in the broader ongoing immunology case-cohort sub-study which randomly selected 45% of all Kilifi participants and 10% of Eldoret study participants as a sub-cohort reflecting survival and mortality in the full REALITY trial. This has now been clarified on page 6.*  

In the methods, the authors say: “The linear regression to examine whether subsequent death and the 3 randomized interventions were associated with the change in plasma calprotectin between baseline and 4 weeks”   The mortality analysis is confusing as written. I don’t think the results were presented in the manuscript? For example, it is not clear what the exposure and outcome variable for this analysis is. If the exposure is change in plasma calprotectin, then the outcome is presumably death after 4 weeks (which would exclude many deaths) and a linear regression does not seem appropriate. But if the exposure is death (i.e. before 4 weeks), then a 4 week sample would not be available.  

*We have revised clarified this in the manuscript in methods on page 9 and results on page 11. In the main analysis, factors associated with plasma calprotectin levels at baseline were assessed using a linear regression model. We have now analysed the associations between change in plasma calprotectin between baseline and 4 weeks and deaths after 4 weeks by calculating relative risk.*

Sample size should be stated for the tables and figures. Was the random subcohort used in any of the presented analysis? E.g. due to random selection, it may be most useful for Table 2 analysis if there are enough cases in the subcohort.  

*The sample size has been added to tables and figures. All analyses include the random sub-cohort and all additional deaths as is usual in a nested case-cohort study.*

Given the small sample size, selecting covariates for multivariable model based on significance on univariate models can present some issues. Lack of significance could be due to the limited power for this specific study although a real association may exist.  

*We agree, and we commented in the discussion that this pilot study should be validated in a larger study.*

It is useful to also see the hazards and 95% CI (not just p-values) in text of results instead of just the table.  

*We have added ß coefficients and hazards ratios to the main text.*

Discussion should include whether this marker could have utility for individuals with less advanced HIV.**

*Thank you, we have added this to the discussion.*

# Peer review: Reviewer response for version 1

**DOI:** 10.21956/wellcomeopenres.17044.r38178

**Published:** 2020-05-01

**Authors:** Rachel A. Silverman

**Affiliations:** 1 Center for Public Health Practice and Research, Department of Population Health Sciences, Virginia Polytechnic Institute and State University, Blacksburg, VA, USA

This study presents results of a pilot study investigating the association between plasma calprotectin at ART initiation with mortality among individuals with advanced HIV. Investigating the use of calprotectin as a prognostic biomarker for advanced HIV could inform its use as a tool in clinical practice to intervene and better prevent early mortality among individuals with advanced HIV initiating ART. The results suggest that plasma calprotectin was associated with mortality within 4 week and within 24 weeks. The case cohort design allowed the researchers to investigate this question accurately without needing samples analyzed from the entire REALITY Trial. The objectives of this pilot study were clearly stated and their results suggest calprotectin could be useful in addressing early mortality among those with advanced HIV initiating ART, an important need when many individuals continue to be diagnosed with HIV late in disease progression due to an opportunistic infection or other late-stage HIV related complication. However, to ensure this article is scientifically sound, there were several areas that could use more clarity or editing. Specifically, there were several results that were lacking associated methods or could use additional clarity. My comments are outlined in order by section below and include both major comments to be addressed in revisions and minor comments related to formatting/typos.

**Abstract:**

**Background:** 

 Major comments: 

- Please specify how “ART initiation” is defined? Is the plasma collected prior to receiving any medication?

**Methods:**

 Minor comment: 

- Space in Trial ID number before closing parentheses.

Major comments:

- Please clarify how non-cases were selected. It states non-cases were randomly selected among those who survived for 48 weeks. Should this state for at least 48 weeks? See additional comments below for the main body methods section.
- The final sentence “To test association with mortality…” is confusing. Suggest rewording. Also, how was clustering by CD4 count decided/done?

**Results:**

 Major comments: 

- The authors described variables associated with baseline calprotectin, but this analysis is not described in the methods. Please add methods for these results in the methods section and explain why this was done. 
- Results mention deaths within 4 weeks as an outcome, but this is not include in the methods. Please ensure all results have methods described.

**Introduction:**

 No comments

**Methods:**

**Study design:**

 Major comments:

- Please clarify why there were 45% randomly selected from Kilfi but only 10% randomly selected from Eldoret? How were these percentages determined for selection?

Minor comment:

- Please define “baseline” in the context of calprotectin samples collected at baseline and 4 weeks after treatment initiation. So baseline is prior to ART initiation, yes? Please clarify. 

**Statistical Analysis:**

 Major comments:

- Please clarify the linear regression analysis. Was the linear regression model was univariable or was multivariable? This seems to be describing stepwise model building, yes? It states that the authors only retained those variables which were statistically significant. How was the order of variable addition determined? Generally, model building should not rely solely on statistical significance. Variables should be determined on the basis of plausibility a priori as well. A variable may be associated with an outcome, but especially in a small pilot, this might not be statistically significant due to insufficient power, so significance should not be the determining factor for its inclusion in the final model. Important to look at point estimates in addition to p-values and known relationships between the variables in identifying confounders to include in subsequent adjusted analyses.
- Please clarify in the Cox proportional hazard regression, how was this stratified by CD4 count. What strata were used and how were these determined?
- Please clarify the following in relation to survival time: the abstract states cases died within 24 weeks and non-cases were those who survived for 48 weeks. Manuscript states time at risk was 24 weeks and there is no mention of 48 weeks. What about those who died between 24 and 48 weeks? And the results present 4 weeks as an outcome end point. Please ensure all results have associated methods and that the Abstract has consistent language with the manuscript main body. 
- Why was the Wilcoxon paired sign-rank test was performed (final paragraph of Statistical Analysis). Please explain the objective/purpose of this analysis and how the covariates included in the model were determined.

**Results**

**Characteristics of study participants:**

 Minor comments:

- Is enrollment misspelled (enrolment)?
- Final sentence is grammatically incorrect. I think deleting the “lower” before “hemoglobin”would correct this.

**Baseline plasma calprotectin:**

 Minor comments: 

- Please define what “crude” means.

Major comments:

- The following language should be adjusted to reduce reliance on p-values for scientific interpretation: “other putative markers of infection (CD8+ T cells, fever, tuberculosis, body temperature, diarrhoea, and cryptococcal antigen test) *did not emerge *as factors affecting plasma calprotectin levels.” Please change this language to specify these were not “statistically significant.” This could be a result of low power. The point estimates should also be discussed, especially since this is a small pilot. 
- Table 2: 95%CI for site (Eldoret) implies statistical significance (-0.57 to -0.14) but p-value is 0.23. Is this row accurate?
- Table 2: Instead of listing the beta, can you please specifically describe what this beta means instead? This is the difference between mean calprotectin levels for a 1 unit change in the variables listed, right? What is the interpretation of this point estimate?
- Table 3: States model was stratified by CD4 counts. How was this stratified and what were the results of the stratified analysis? What are the strata and how were they selected? This should be discussed in the methods.

**Plasma calprotectin after 4 weeks:**

- Please show or at least describe the following data: “There was no evidence that any of the three randomized interventions affected the change in calprotectin between baseline and 4 weeks (data not shown).” Rephrase to describe lack of statistical significance and also discuss the point estimates.

**Discussion:**

 Minor comment:

- The following could be clarified: “This pilot study focused on assessing plasma calprotectin mortality among patients with advanced HIV disease determined by a very low CD4+ cell count.” Reword to include more detail: “This pilot study focused on assessing the association with pre-ART plasma calprotectin and mortality within 24 weeks among patients with advanced HIV disease defined by a very low CD4+ cell count (<100cells/µl).”

Major comments:

- The following is confusing: “It appeared that calprotectin levels at ART initiation are likely to be predictive of deaths occurring in the first 4 weeks.” Please use more concrete language to describe your main finding. Also, it is unclear if 24 weeks or 4 weeks is your survival end point. Calprotectin levels were associated with both, right?
- Please describe your limitations specifically. “However, the sample size for this exploratory analysis was limited. This pilot study requires validation in a larger sample and in other clinical settings before being used to guide further investigations or specific interventions.” Should be explicit about your limitations, how they may have impacted your results, and thus why next steps are needed. You do this at the end of the discussion section, so confusing why you mention this in two places. This is also the first time the term “exploratory” is used in the manuscript. Please include this in the methods so it’s clear what you mean by this term.
- Please adjust language in the following: “However, markers of infection markers apart from cough were not associated with baseline plasma calprotectin.” The phrase “not associated” is not ideal. Better to state: “However, markers of infection markers apart from cough were not statistically significantly associated with baseline plasma calprotectin.” And discuss the point estimate. Why do you think cough associated but others not? Is this just an issue of multiple comparisons and a spurious association or do you have insufficient power to detect other infection markers? I think this is worth further discussion. 
- The authors mention “…potential for bias during sample selection as included participants were required to have a full set of sample” but you state earlier that you are confident this was missing at random, so this is assumed not to have biased your results, right? Please include this information here if you are confident in the MAR assumption and it’s impact on your analysis. 
- Can you please discuss if it will be feasible for the ELISA test to be used in low/middle income locations where it could be of most benefit due to high rates of ART initiation at an advanced HIV stage? Do you think ART clinics have the infrastructure and resources to perform this test to inform patient care or are costs and lab resources potentially another barrier?

Is the work clearly and accurately presented and does it cite the current literature?

Yes

If applicable, is the statistical analysis and its interpretation appropriate?

Partly

Are all the source data underlying the results available to ensure full reproducibility?

Partly

Is the study design appropriate and is the work technically sound?

Partly

Are the conclusions drawn adequately supported by the results?

Partly

Are sufficient details of methods and analysis provided to allow replication by others?

Partly

Reviewer Expertise:

Epidemiology, HIV treatment and prevention, mortality following ART initiation. This topic was a focus of my recent dissertation work, though I have since switched to other topics related to other STDs and specific issues related to health in Southwest Virginia in the United States, so my knowledge on this specific topic may be a couple of years out of date.

I confirm that I have read this submission and believe that I have an appropriate level of expertise to confirm that it is of an acceptable scientific standard, however I have significant reservations, as outlined above.

# Response

**Published:** 2020-09-29

**Authors:** James Berkley

We thank the reviewer for their comments providing an opportunity to improve our manuscript. Please find out point-by-point responses below.  

**Reviewer 1** Please specify how “ART initiation” is defined? Is the plasma collected prior to receiving any medication?  

*Yes, plasma was collected before any ART was given. This has been added on page 7.* 

Space in Trial ID number before closing parentheses.  

*Thank you, corrected.*  

Please clarify how non-cases were selected. It states non-cases were randomly selected among those who survived for 48 weeks. Should this state for at least 48 weeks? See additional comments below for the main body methods section.  

*We randomly selected participants who were confirmed to have survived to the end of the trial at 48 weeks (non-cases) for whom samples were available. This has been clarified on page 8.*     

The final sentence “To test association with mortality…” is confusing. Suggest rewording. Also, how was clustering by CD4 count decided/done?  

*We three strata for the CD4 ^+^ count groups(0-24, 25-49, and 50-99 cells/mm ^3^) as per the sampling frame. The has been revised for clarity n page 8 and the final text of the section also refers to this.*  

The authors described variables associated with baseline calprotectin, but this analysis is not described in the methods. Please add methods for these results in the methods section and explain why this was done.  

*Associations with plasma calprotectin at baseline were assessed using linear regression, this is described in the second paragraph under Statistical methods.*

esults mention deaths within 4 weeks as an outcome, but this is not include in the methods. Please ensure all results have methods described.  

*Thank you, this has been added to the bottom of page 8.*  

Please clarify why there were 45% randomly selected from Kilfi but only 10% randomly selected from Eldoret? How were these percentages determined for selection?  

*The broader case cohort study involved two of the sites in the broader ongoing immunology case-cohort sub-study which randomly selected 45% of all Kilifi participants and 10% of Eldoret study participants as a sub-cohort reflecting survival and mortality in the full REALITY trial. This has now been clarified on page 6.*  

Please define “baseline” in the context of calprotectin samples collected at baseline and 4 weeks after treatment initiation. So baseline is prior to ART initiation, yes? Please clarify.

*Calprotectin was measured using samples collected at baseline, before ART treatment was initiated, and four weeks after ART treatment initiation. We have clarified this on page 7.* 

Please clarify the linear regression analysis. Was the linear regression model was univariable or was multivariable? This seems to be describing stepwise model building, yes? It states that the authors only retained those variables which were statistically significant. How was the order of variable addition determined? Generally, model building should not rely solely on statistical significance. Variables should be determined on the basis of plausibility a priori as well. A variable may be associated with an outcome, but especially in a small pilot, this might not be statistically significant due to insufficient power, so significance should not be the determining factor for its inclusion in the final model. Important to look at point estimates in addition to p-values and known relationships between the variables in identifying confounders to include in subsequent adjusted analyses.  

*We have now clarified on page 8 and table 2 that the linear regression model was stepwise, always including age, sex, site, CD4+, viral load and neutrophil count, and selecting other biologically plausible variables on the basis of statistical significance at P<0.1. We regarded that including all of the tested variables in the final model *     

Please clarify in the Cox proportional hazard regression, how was this stratified by CD4 count. What strata were used and how were these determined?  

*We used three strata for the CD4 ^+^ count groups(0-24, 25-49, and 50-99 cells/mm ^3^) as per the sampling frame. The has been revised for clarity n page 8 and the final text of the section also refers to this. In STATA, the command was ‘stcox ... , strata(cd4group)’.* 

Please clarify the following in relation to survival time: the abstract states cases died within 24 weeks and non-cases were those who survived for 48 weeks. Manuscript states time at risk was 24 weeks and there is no mention of 48 weeks. What about those who died between 24 and 48 weeks? And the results present 4 weeks as an outcome end point. Please ensure all results have associated methods and that the Abstract has consistent language with the manuscript main body.  

*Thank you. This is an error, the parent trial primary endpoint was mortality to 24 weeks, with secondary outcomes including those during extended follow up to 48 weeks, but for this analysis, survival or death during 24 weeks was used. This has been corrected through the manuscript. . We have added the 4-week analysis to the methods.*     

Why was the Wilcoxon paired sign-rank test was performed (final paragraph of Statistical Analysis). Please explain the objective/purpose of this analysis and how the covariates included in the model were determined.**

*The Wilcoxon paired sign-rank test was used to assess the overall change in plasma calprotectin between baseline and after four weeks, without covariates.*  

Is enrollment misspelled (enrolment)?  

*English (United Kingdom) spelling was used since this is a UK-based journal.*     

Final sentence is grammatically incorrect. I think deleting the “lower” before “hemoglobin”would correct this.  

*Thank you. We have revised as suggested.*  

Please define what “crude” means.  

*We have replaced ‘crude’ with ‘unadjusted’.*  

The following language should be adjusted to reduce reliance on p-values for scientific interpretation: “other putative markers of infection (CD8+ T cells, fever, tuberculosis, body temperature, diarrhoea, and cryptococcal antigen test) did not emerge as factors affecting plasma calprotectin levels.” Please change this language to specify these were not “statistically significant.” This could be a result of low power. The point estimates should also be discussed, especially since this is a small pilot.  

*We have revised this to indicate P<0.1 was used for selection. We agree with the issue of reliance on P-values but regarded that having many variables in the final model was undesirable in a small pilot.   *     

Table 2: 95%CI for site (Eldoret) implies statistical significance (-0.57 to -0.14) but p-value is 0.23. Is this row accurate?  

*Thank you. It has been corrected to -0.57 to 0.14. *     

Table 2: Instead of listing the beta, can you please specifically describe what this beta means instead? This is the difference between mean calprotectin levels for a 1 unit change in the variables listed, right? What is the interpretation of this point estimate?  

*We have added the interpretations to table 2.*     

Table 3: States model was stratified by CD4 counts. How was this stratified and what were the results of the stratified analysis? What are the strata and how were they selected? This should be discussed in the methods.  

*We used three strata for the CD4 ^+^ count groups (0-24, 25-49, and 50-99 cells/mm ^3^) as participants were selected using these strata to avoid imbalance in a key exposure (as per https://pubmed.ncbi.nlm.nih.gov/10763560/). This is added to page 6. All analyses are stratified.* 

Please show or at least describe the following data: “There was no evidence that any of the three randomized interventions affected the change in calprotectin between baseline and 4 weeks (data not shown).” Rephrase to describe lack of statistical significance and also discuss the point estimates.  

*These estimates have been added on page 11 and discussed on page 13.*  

The following could be clarified: “This pilot study focused on assessing plasma calprotectin mortality among patients with advanced HIV disease determined by a very low CD4+ cell count.” Reword to include more detail: “This pilot study focused on assessing the association with pre-ART plasma calprotectin and mortality within 24 weeks among patients with advanced HIV disease defined by a very low CD4+ cell count (<100cells/µl).” *This has been reworded as suggested.* 

The following is confusing: “It appeared that calprotectin levels at ART initiation are likely to be predictive of deaths occurring in the first 4 weeks.” Please use more concrete language to describe your main finding. Also, it is unclear if 24 weeks or 4 weeks is your survival end point. Calprotectin levels were associated with both, right?  

*Thank you. The primary analysis is to 24 weeks and this has been clarified along with a more concrete statement.*     

Please describe your limitations specifically. “However, the sample size for this exploratory analysis was limited. This pilot study requires validation in a larger sample and in other clinical settings before being used to guide further investigations or specific interventions.” Should be explicit about your limitations, how they may have impacted your results, and thus why next steps are needed. You do this at the end of the discussion section, so confusing why you mention this in two places. This is also the first time the term “exploratory” is used in the manuscript. Please include this in the methods so it’s clear what you mean by this term.  

*Thank you. The term ‘exploratory’ has been removed and we have revised the limitations as suggested.*         

Please adjust language in the following: “However, markers of infection markers apart from cough were not associated with baseline plasma calprotectin.” The phrase “not associated” is not ideal. Better to state: “However, markers of infection markers apart from cough were not statistically significantly associated with baseline plasma calprotectin.” And discuss the point estimate. Why do you think cough associated but others not? Is this just an issue of multiple comparisons and a spurious association or do you have insufficient power to detect other infection markers? I think this is worth further discussion.

*Thank you, we have made this change. Insufficient power is quite possible and we have included that.*     

The authors mention “…potential for bias during sample selection as included participants were required to have a full set of sample” but you state earlier that you are confident this was missing at random, so this is assumed not to have biased your results, right? Please include this information here if you are confident in the MAR assumption and it’s impact on your analysis.  

*We have remove ‘missing at random’ since sites were differentially affected and mentioned this under limitations.*     

Can you please discuss if it will be feasible for the ELISA test to be used in low/middle income locations where it could be of most benefit due to high rates of ART initiation at an advanced HIV stage? Do you think ART clinics have the infrastructure and resources to perform this test to inform patient care or are costs and lab resources potentially another barrier?  

*We have added this to the conclusions: ‘Although capacity for an ELISA-based test may be limited at many clinics, there is at least one quantitative serum calprotectin lateral-flow test available’.*
